# Preparation and Characterization of Nanosuspension of Aprepitant by H96 Process

**DOI:** 10.15171/apb.2016.013

**Published:** 2016-03-17

**Authors:** Sunethra Kalvakuntla, Mangesh Deshpande, Zenab Attari, Koteshwara Kunnatur B

**Affiliations:** ^1^ Department of Pharmaceutics, Manipal College of Pharmaceutical Sciences, Manipal University, Manipal.; ^2^ Dr. Reddy’s Laboratories Ltd., Hyderabad, India.

**Keywords:** Lyophilization, Nanosuspension, Particle size, Second generation approach, High pressure homogenization

## Abstract

***Purpose:*** Nanosuspension in drug delivery is known to improve solubility, dissolution and eventually bioavailability of the drugs. The purpose of the study was to compare particle size of nanosuspensions prepared by the first generation approach and H96 approach and to evaluate the effectiveness of H96 approach.

***Methods:*** The nanosuspension of aprepitant was prepared by HPH and H96 approach. The prepared nanosuspensions were characterized for their particle size and zeta potential. The optimized nanosuspension was further evaluated for DSC, FT-IR, solubility and dissolution.

***Results:*** The optimized nanosuspension (NCLH5) prepared using combination of tween 80 and poloxamer 188 as stabilizer, showed particle size of 35.82 nm and improved solubility and dissolution profile over pure drug. NCLH5 was chosen optimized formulation and further evaluated for other parameters after lyophilization. Lyophilization resulted in increase in particle size. The solubility and dissolution studies showed favorable increase in the performance. The FT-IR and DSC analysis showed change in the crystallinity after nanosizing.

***Conclusion:*** The observations indicated that lyophilization prior to high pressure homogenization resulted in efficient particle size reduction yielding smaller particles than first generation preparation technique. H96 is a good and easy alternative to achieve efficient particle size reduction of drug in lesser time and increase its solubility and dissolution.

## Introduction

Significant progress has been made in the area of supportive care in oncology over the last decade as chemotherapy-induced nausea and vomiting (CINV) has been a major problem leading to patients’ refusal to continue chemotherapy. Aprepitant is an orally active NK 1 (neurokinin 1) receptor antagonist, used for the treatment of CINV.^1,2^

Aprepitant is given in combination with ondansetron and dexamethasone on day 1 and then continued on days 2 and 3 with dexamethasone which consequently improves acute, as well as delayed chemotherapy associated emesis. Aprepitant is a BCS class IV drug, having low solubility, low permeability).The bioavailability of aprepitant is dissolution rate limited following oral administration.^3,4^

Drug nanocrystals are a formulation approach to improve solubility of poorly soluble drugs and cosmetic actives. It has been first invented at the beginning of the 1990s and the first pharmaceutical product came in the year 2000. Arbitrarily, two generations of nanocrystals are proposed depending on the methods of preparation or technique used. The nanocrystal technology of the first generation comprises ball milling or high pressure homogenization (HPH) as a method of preparation.^5^ SmartCrystals are the second generation nanocrystals prepared by the combination of methods. The production of smart crystals has been optimized by introducing modifications to the HPH process. This leads to faster production, smaller nanocrystals and an improved physical stability. This has also implications for improved *in vivo* performance after dermal application and oral or intravenous administration.^6^

In the previous study, we prepared nanosuspension of ibuprofen and aprepitant using combination of precipitation or ball milling with high pressure homogenization (HPH) and observed reduction in particle size with less processing time.^7^ In present study two approaches i.e. HPH technique (first generation) and combination of lyophilization and HPH (second generation; H96 process) were evaluated for the production of aprepitant nanocrystals to enhance solubility and dissolution for enhancing bioavailability and reducing variability in systemic exposure.

## Materials and Methods

The drug, aprepitant was provided as a kind gift by Dr. Reddy’s Laboratories Ltd. All the chemicals and reagents used in the present study were of analytical grade. Tween 80 was procured from National Chemicals, Polyvinyl alcohol (PVA – Mw: 14000) from SD Fine-Chem. Ltd. and sodium lauryl sulfate (SLS) from Nice Chemicals Pvt. Ltd.

### Preparation of nanosuspensions

#### High pressure homogenization (HPH)

Aqueous solutions of stabilizers i.e. Tween 80, Poloxamer 188, PVA and SLS were prepared in various concentrations as shown in the Table 1 using purified water. Aprepitant (125 mg) was suspended in 10 ml of the stabilizer solution. The dispersion was homogenized using high speed homogenizer (Polytron PT 3100, Kinematica) at 10,000 rpm for 10 min to form homogeneous microsuspension. This was subjected to probe sonication (Vibracell VCX130; Sonis, USA) at amplitude of 80%, pulse 4 sec for 15 min to form presuspension. During this sonication, the temperature was maintained at 0°C using an ice bath. This presuspension was added dropwise to the remaining stabilizer solution and homogenized. Firstly, premilling step was conducted at 5000 psi for 5 cycles using high pressure homogenizer (Emulsiflex-C3, Avestin, USA). Then an HPH step was applied at 15000 psi for 10 cycles.

**Table 1:** Formulation batches by a) high pressure homogenization technique (First generation approach) and b) H96 process (Lyophilization + HPH)

**Formulation code**	**Formulation Composition**					
	**Aprepitant** **(mg)**	**Tween 80** **(%w/v)**	**Poloxamer 188 (%w/v)**	**PVA (%w/v)**	**SLS (%w/v)**	**Batch Size (ml)**
a) Formulation batches by high pressure homogenization technique (First generation approach)						
**NCH1**	125	0.25	-	-	-	40
**NCH2**	125	0.5	-	-	-	40
**NCH3**	125	1	-	-	-	40
**NCH4**	125	2	-	-	-	40
**NCH5**	125	3	-	-	-	40
**NCH6**	125	-	0.25	-	-	40
**NCH7**	125	-	0.5	-	-	40
**NCH8**	125	-	1	-	-	40
**NCH9**	125	-	2	-	-	40
**NCH10**	125	-	3	-	-	40
**NCH11**	125	-	-	0.25	-	40
**NCH12**	125	-	-	0.5	-	40
**NCH13**	125	-	-	1	-	40
**NCH14**	125	-	-	2	-	40
**NCH15**	125	-	-	3	-	40
**NCH16**	125	-	-	-	0.25	40
**NCH17**	125	-	-	-	0.5	40
**NCH18**	125	-	-	-	1	40
**NCH19**	125	-	-	-	2	40
**NCH20**	125	-	-	-	3	40
**NCH21**	125	1	3	-	-	40
**NCH22**	125	1	-	0.25	-	40
**NCH23**	125	1	-	-	3	40
b) Formulation batches by H96 process (Lyophilization + HPH)						
**NCLH1**	125	1	-	-	-	40
**NCLH2**	125	-	3	-	-	40
**NCLH3**	125	-	-	0.25	-	40
**NCLH4**	125	-	-	-	3	40
**NCLH5**	125	1	3	-	-	40
**NCLH6**	125	1	-	0.25	-	40
**NCLH7**	125	1	-	-	3	40

#### H96 (lyophilization + HPH)

The H96 process is a combination of lyophilization and HPH techniques. The amount of drug and organic solvent are crucial factors to be considered for lyophilization in H96 process.^8^ In the last step of drug (aprepitant) synthesis, no crystallization of the drug is performed but the drug solution was made using methanol as solvent. Methanol was solvent of choice as aprepitant was observed to be freely soluble in it. The prepared drug solution was then lyophilized. In the next step, the lyophilized product was dispersed in a various stabilizers solutions of concentrations (shown in the Table 1) using purified water, which was immediately passed through a homogenizer 15000 psi for 5 cycles. Four different stabilizers (Tween 80, Poloxamer 188, PVA and SLS) were screened in different concentrations, alone and in combinations (Table 1). The concentration of stabilizers was selected from the results obtained in HPH (first generation) process.

### Shape and surface morphology: Scanning Electron Microscopy (SEM)

Immediately after freeze drying, the dry powder was examined for possible aggregation by visual inspection. Shape and surface morphology of the freeze dried nanocrystals was studied using SEM (JEOL, JSM 50A, Tokyo, Japan). An appropriate amount of freeze dried nanocrystals was mounted on metal (aluminium) stubs; the samples were mounted onto aluminium specimen stubs using double-sided adhesive tape and fractured with a razor blade. The samples were sputter-coated with gold/palladium for 120 sec at 14 mA under argon atmosphere for secondary electron emissive SEM and observed for morphology, at acceleration voltage of 20 KV.

### Particle size and size distribution

The particle size and its distribution were determined using Zetasizer Nano ZS (Malvern instruments, U K) using a process called Dynamic Light Scattering (DLS). The zeta potential of a particle is the overall charge that the particle acquires in a particular medium. The particle size and zeta potential of nanosuspension samples were measured at 25°C. The nanosuspension by combination method showing the lowest particle size with acceptable zeta potential was selected for further studies. Though the zeta potential indicates the stability of the nanosuspension, however, the freeze drying is good for long term stability of colloidal nanoformulations and thus the optimized nanosuspensions were lyophilized using mannitol as cryoprotectant and subjected to various evaluation.^9^

### Drug content

The drug content in the freeze dried product was analyzed by dissolving 10 mg of lyophilized nanocrystals in 10 ml of methanol. The sample was sonicated for 15 minutes and filtered using 0.22 μ membrane filter and after sufficient dilution, the amount of drug was determined spectrophotometrically at 210 nm (UV 1601PC, Shimadzu, Japan). The UV method was selected by scanning lower concentrations of the aprepitant in various media (methanol, pH 1.2 HCl buffer, pH 4.6 acetate buffer, pH 6.8 phosphate buffer, pH 7.4 phosphate buffer, distilled water, 2.2% SLS) to find out the maximum wavelength after nullifying the interference of the media.^10^

### Saturation solubility 

An excess amount of the freeze dried product was added separately to 4 ml each of distilled water, pH 1.2 HCl buffer + 2.2% w/v SLS, pH 4.6 acetate buffer + 2.2% w/v SLS, pH 6.8 phosphate buffer, pH 7.4 phosphate buffer, 2.2% w/v SLS solution. Then the mixtures were mounted on rotospin apparatus for 48 hrs at room temperature. The solution was filtered through a 0.22 μ membrane filter and the amount of the drug dissolved was analyzed using spectrophotometer at 210 nm.

### Fourier-transform infra red spectroscopy (FT-IR) and differential scanning calorimetry (DSC)

The optimized formulation was subjected to FT-IR (Shimadzu FT-IR 8300 spectrophotometer) and DSC (DSC-60, Shimadzu, Japan) analysis and compared with that of pure drug to assess drug-excipient interaction and crystallinity of the drug in the formulation.

### In vitro release studies

The *in vitro* dissolution study of nanocrystals and pure drug was carried out in 900 ml of different discriminating media such as 2.2% w/v SLS solution, pH 1.2 HCl buffer + 2.2% w/v SLS, pH 4.6 acetate buffer + 2.2% w/v SLS, pH 6.8 phosphate buffer + 2.2% w/v SLS, pH 7.4 phosphate buffer + 2.2% w/v SLS using USP type I dissolution apparatus at 75 rpm (US FDA dissolution methods).^11^ The temperature of the dissolution medium was maintained at 37±0.5°C by a thermostatically controlled water bath. Five ml of sample was withdrawn at the time intervals of 10, 15, 20, 30 and 45 minutes and replaced with 5 ml of fresh buffer. The collected sample was filtered and analyzed spectrophotometrically at 210 nm. The dissolution profile of nanocrystal was compared with that of pure drug. The results were analyzed using student’s t-test.

## Results

### Preparation and characterization of nanosuspension

The particle size of various batches of nanosuspension prepared by HPH perse and H96 process is depicted in Table 2. The stabilizers, Tween 80 and Poloxamer 188 resulted in smaller particle size compared to PVA and SLS. The combination of Tween 80 and Poloxamer 188 was observed to be effective in stabilization of prepared nanpsuspension. H96 process, i.e. combination of lyophilization and HPH resulted in smaller particle size than HPH perse. The particle size of NCH21 prepared by HPH perse using Tween 80 and Poloxamer 188 was found to be 320.4 nm whereas, NCLH5 prepared by H96 process using same stabilizers was observed to be 35.78 nm. The particle size of NCLH5 is smaller and thus chosen as optimized formulation, however, lyophilization resulted in increase in particle size. The particle size and zeta potential of the NCLH5 before and after lyophilization was depicted in the Table 3.

### Shape and surface morphology: Scanning Electron Microscopy (SEM) 

**Figure 1:**
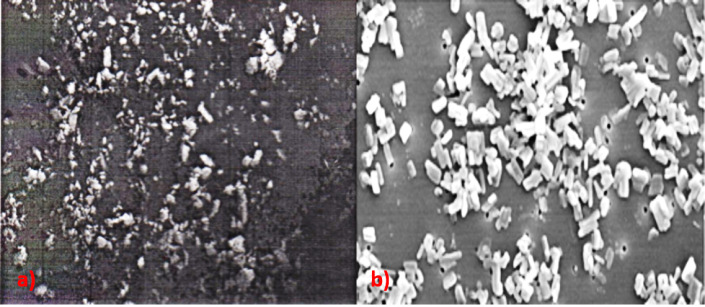
SEM images of a) pure drug and b) aprepitant nanocrystals

The freeze dried optimized nanocrystals (NCLH5) was observed for the shape and surface morphology by SEM. The SEM images of pure drug showed agglomerates (a) and that of the optimized formulation showed the particles are discrete without agglomeration (b) which could be attributed to the presence of stabilizer (Figure 1).

The SEM images confirmed that though an increase in particle size was observed after freeze drying, it was still in the submicron level and smaller in size in comparison with pure drug, but not below 100 nm (smart crystal).

**Table 2:** Particle size and zeta potential of nanosuspensions prepared by a) HPH process (First generation approach) and b) H96 process (Lyophilization + HPH)

**Formulation**	**Particle size (nm)**	**PDI**	**ZP (mV)**
a) Particle size and zeta potential of nanosuspensions prepared by HPH process (First generation approach)			
**NCH1**	516.2	0.293	-18.60
**NCH2**	443.2	0.218	-22.93
**NCH3**	433.1	0.288	-36.79
**NCH4**	373.9	0.306	-23.26
**NCH5**	351.0	0.278	-29.26
**NCH6**	840.9	0.331	-13.90
**NCH7**	815.8	0.294	-25.10
**NCH8**	890.7	0.298	-25.80
**NCH9**	784.6	0.269	-21.80
**NCH10**	840.0	0.256	-22.80
**NCH11**	960.2	0.227	-2.20
**NCH12**	1011.9	0.594	-6.61
**NCH13**	1169.4	0.369	-1.92
**NCH14**	1339.3	0.321	-2.37
**NCH15**	1502.9	0.341	-8.21
**NCH16**	1132.6	0.243	-8.41
**NCH17**	870.5	0.252	-6.21
**NCH18**	763.8	0.215	-4.08
**NCH19**	698.2	0.189	-13.64
**NCH20**	678.2	0.165	-10.22
**NCH21**	320.4	0.266	-28.80
**NCH22**	526.2	0.293	-15.10
**NCH23**	510.5	0.272	-16.92
b) Particle size and zeta potential of nanosuspensions prepared by H96 process (second generation approach)			
**NCLH1**	89.1	0.288	-20.60
**NCLH2**	125.1	0.141	-16.93
**NCLH3**	260.6	0.274	-5.79
**NCLH4**	178.7	0.255	-6.26
**NCLH5**	**35.78**	**0.257**	**-23.2**
**NCLH6**	126.5	0.181	-13.90
**NCLH7**	110.8	0.159	-18.10

**Table 3:** Particle size and zeta potential of optimized formulation before and after lyophilization

**Optimized formulation (NCLH5)**	**Before lyophilization**	**After lyophilization**
**Particle size (nm)**	35.78	119.9
**Zeta potential (mV)**	-23.2	-32.4

### Drug content

The drug content of the freeze dried formulation (NCLH5) was found to be around 90%. The loss of drug can be attributed to the loss occurring during the preparation and lyophilization.^12^ However, there was no change in color or aggregation observed.

### Saturation Solubility

Saturation solubility study was carried out for both pure drug and nanocrystals in distilled water, pH 1.2 HCl buffer + 2.2% w/v SLS, pH 4.6 acetate buffer + 2.2% w/v SLS, phosphate buffer pH 6.8, phosphate buffer pH 7.4, 2.2% w/v SLS solution (as per FDA guidelines, pH 1 to 7.4).^13^ However, it was found that drug was getting precipitated in HCl buffer pH 1.2 and acetate buffer pH 4.6, therefore, 2.2% w/v SLS was added to both the buffers. The saturation solubility of aprepitant was observed to increase over pure drug in all the vehicles used. This is due to the decrease in particle size when compared to pure dug according to Ostwald-Freundlich equation. Another possible explanation for the increase in saturation solubility can be given by Kelvin equation which states that the dissolution pressure increases with increasing curvature, which means decreasing particle size.^14^ The curvature is enormous when the particle size is in the nanometer range. The fold increase in the solubility of nanocrystal over pure drug is depicted in Table 4.

**Table 4:** Saturation Solubility of pure drug and aprepitant nanocrystals

**Medium**	**Pure drug Concentration (µg/ml) (Mean ± SD)**	**Nanocrystals Concentration (µg/ml) (Mean ± SD)**	**Fold increase in saturation solubility**
Distilled water	2.90 ± 0.53	43.1 ± 5.10	14.86
pH 1.2 buffer + 2.2%w/v SLS	2736 ± 30.38	3314 ± 10.99	1.21
pH 4.6 buffer + 2.2%w/v SLS	3364 ± 12.59	3854 ± 8.17	1.15
Phosphate buffer pH6.8	3.4 ± 2.38	38.6 ± 3.94	11.35
Phosphate buffer pH7.4	3.52 ± 1.22	40.2 ± 5.10	11.42
2.2%w/v SLS in water	3910 ± 31.40	4610 ± 7.21	1.18

### FT-IR and DSC

**Figure 2:**
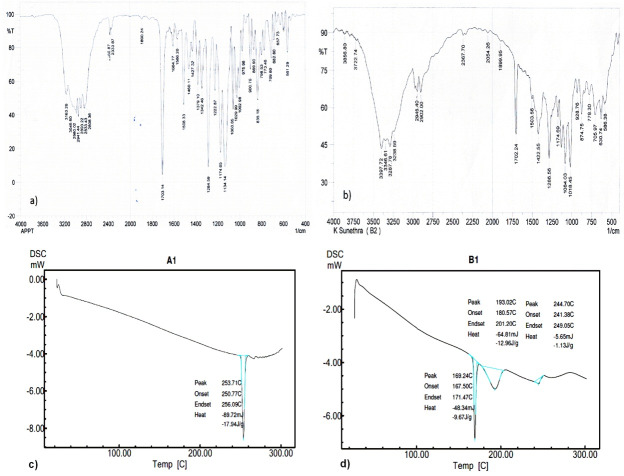
FT-IR spectra of a) aprepitant (pure drug) and b) aprepitant nanocrystal and DSC thermograms of c) aprepitant (pure drug) and d) aprepitant nanocrystal

In the case of formulation NCLH5, disappearance of two peaks in the FT-IR spectrum was observed along with the attenuation of other peaks when compared to that of pure drug which may be due to the reduction in crystallinity of the drug (Figure 2).^15^

The assessment crystalline state helps in understanding the polymorphic changes that the drug might have undergone when subjected to nanosizing. So it is necessary to investigate the extent of amorphous state generated during the production of nanosuspensions. In DSC thermograms, pure drug showed an intense peak at 253.71°C whereas the drug peak in nanoformulation was observed at 244.70°C. The heat of melting was observed to be -17.94 J/g for pure drug and -1.13 J/g for nanoformulation. The shift in the peak and reduction in the peak intensity indicated a change in the crystallinity of the drug (Figure 2). The other reason could be presence of large amount of excipient, mannitol.^16^

### In vitro dissolution studies

**Figure 3:**
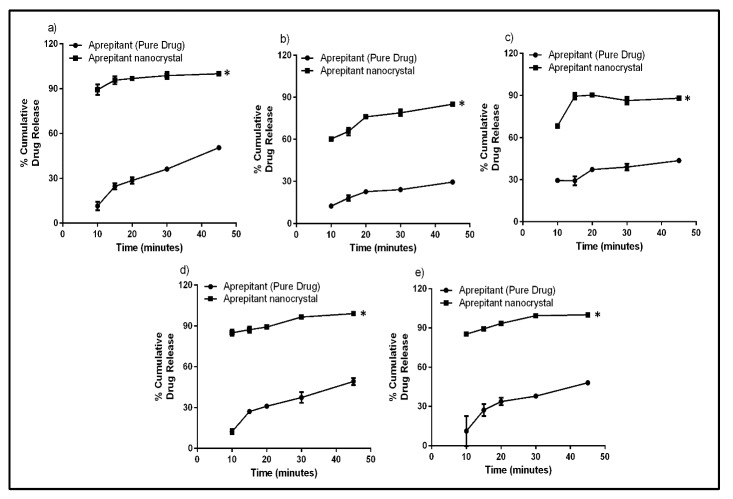
Dissolution profile of pure drug and aprepitant nanocrystal in various media a) 2.2% SLS, b) pH 1.2 HCl buffer + 2.2% SLS, c) pH 4.6 acetate buffer + 2.2% SLS, d) pH 6.8 phosphate buffer + 2.2% SLS and e) pH 7.4 phosphate buffer + 2.2% SLS
(* indicates significant difference between two groups at p<0.05 using student’s t-test.)

The *in vitro* dissolution studies were carried out in different media. 2.2% SLS was added in all the buffers to improve solubility of drug and avoid precipitation of drug. It was observed that dissolution of aprepitant crystal was significantly higher than the pure drug irrespective of the medium used. The two times fold increase in dissolution of the nanocrystals as compared to pure drug was observed at 45 minutes (see Table 5) and the dissolution profile of pure drug and nanocrystal are depicted in Figure 3.

**Table 5:** Percentage enhancement of dissolution of aprepitant nanocrystals as compared to pure drug at 45 minutes

**Dissolution medium**	**%CDR at 45 minutes (Mean±SD)**		**Fold enhancement of dissolution of nanocrystal**
	**Pure drug**	**Nanocrystals**	
2.2%w/v SLS	50.52 ± 1.05	100.05 ± 0.53	1.98
pH 1.2 HCl buffer + 2.2% w/v SLS	29.52 ± 0.55	85.05 ± 0.75	2.88
pH 4.6 acetate buffer + 2.2% w/v SLS	43.59 ± 0.55	88.05 ± 1.75	2.01
pH 6.8 phosphate buffer + 2.2% w/v SLS	49.18 ± 2.55	99.03 ± 1.83	2.01
pH 7.4 phosphate buffer + 2.2% w/v SLS	48.11 ± 1.13	100.93 ± 0.65	2.09

## Discussion

In first generation approach i.e. HPH, the stabilizers and its concentration were observed to greatly influence the particle size of the nanosuspensions. In case of PVA, it was observed that the particle size increased as the amount of stabilizer increased. While in case of Poloxamer 188 there was no significant change in particle size occurred with increase in the stabilizer concentration. In presence of Tween 80, particle size decreased with increase in concentration. Various reports suggest that combination of two stabilizers, particularly one surfactant stabilizer and one polymeric stabilizer gives more thermodynamically stable nanosuspensions.^17^ Therefore, Tween 80 (surfactant stabilizer) was coupled with different polymeric stabilizers viz. PVA, SLS and Poloxamer 188 to achieve desired stability. The concentration of Tween 80 was fixed to 1%w/v as it was found that zeta potential of NCH3 was -36.79 mV which is expected to be a stable formulation according to the literature.^18^

The selected batches from first generation approach using specific concentration of stabilizers, which showed optimum particle size, were subjected to second generation process (H96 process). The particle size, PDI and zeta potential of nanosuspensions prepared by H96 process were summarized in Table 4. The particle size of the nanosuspension batch, NCLH1 using Tween 80 as stabilizer was found to be 89 nm (lower than 100 nm) a PDI of 0.288. The zeta potential was observed to be -20.60 indicating its stability however; sedimentation was seen after 24 hours. The particle size of nanosuspension with Poloxamer 188 was observed to be 125.1 nm (slightly above 100 nm) with a low PDI of 0.141. However, the zeta potential of -16.93 mV did not indicate its stability. In addition the sedimentation was also seen. The particle size of nanosuspensions with PVA (NCLH3) and SLS (NCLH4) was not even near to 100 nm and the nanosuspension was not stable. Thus, combination strategy using one surfactant stabilizer and one polymeric stabilizer was attempted here to improve the stability of the nanosuspensions. Tween 80 (surfactant stabilizer) was coupled with different polymeric stabilizers viz. PVA, SLS and Poloxamer 188 to achieve desired stability. Concentration of Tween 80 was fixed to 1%w/v as it showed highest stability in HPH process.

The use of Poloxamer 188 and Tween 80 (NCLH5), gave a particle size of 35.78 nm (Smart Crystals) with a PDI of 0.257 and an acceptable zeta potential of -23.2 mV (Figure 1). Earlier, Moschwitzer (2006) filed a patent for amphotericin B nanosuspension with 62 nm particle size prepared by H96 process.^19^ Moschwitzer lyophilized the drug using liquid nitrogen, however, in the present case; the drug was lyophilized by storing at -80°C followed by freeze drying. The use of Tween 80 with PVA (NCLH6) and SLS (NCLH7) resulted in particle size near to 100 nm. These batches had a low PDI compared to NCLH5 but, their zeta potential was not indicating any stability. Hence, NCLH5 was chosen as optimized formulation and lyophilized for further studies. Although lyophilized powder was free flowing, there was significant increase in particle size which needs attention to overcome this (Table 5 and Figure 2).

H96 process led to efficient size reduction, eventually resulting in smaller particles of drug (aprepitant in our case) in nanosuspension than conventional approach (as reported by Salazar 2011).^20^ It has been reported that lyophilization of drug dispersed in organic solvent lead to physical modification of drug particle which possess advantage of uniformly dispersed coarse drug in stabilizer solution and reduction in particle with less processing time and wear-tear effect.^21^ We also observed that smaller particle size (35.78 nm) was obtained in 5 cycles of homogenization (lesser time than first generation approach - 10 cycles) and the PDI of 0.257 indicated uniform particle size distribution in nanosuspension.

The selected nanoformulation, NCLH 5 showed acceptable nanosized particles in SEM and by zetasizer and exhibited improved solubility and dissolution profile over pure drug in different media. These observations suggested that combination of size reduction methods, lyophilization with HPH in the present case, can be successfully employed to improve solubility of drugs or active compounds in lesser time. Furthermore, it needs to be evaluated for its application in large scale production of different dosage forms.

## Acknowledgments

The authors are thankful to Manipal university for providing the facilities to carry out the present work and also would like to thank Dr. Reddy’s Laboratories Ltd. for providing us the drug.

## Ethical Issues

Not applicable.

## Conflict of Interest

The authors declare no conflict of interest.

## Abbreviations

CINV – chemotherapy induced nausea and vomiting; NK 1 – neurokinin 1; BCS – biopharmaceutics classification system; HPH – high pressure homogenization; SEM – scanning electron microscopy; DLS – dynamic light scattering; FT-IR – fourier-transform infra red; DSC – differential scanning calorimetry

## References

[1] Chawla SP, Grunberg SM, Gralla RJ, Hesketh PJ, Rittenberg C, Elmer ME (2003). Establishing the dose of the oral nk1 antagonist aprepitant for the prevention of chemotherapy-induced nausea and vomiting. Cancer.

[2] Hesketh PJ, Grunberg SM, Gralla RJ, Warr DG, Roila F, de Wit R (2003). The oral neurokinin-1 antagonist aprepitant for the prevention of chemotherapy-induced nausea and vomiting: A multinational, randomized, double-blind, placebo-controlled trial in patients receiving high-dose cisplatin--the aprepitant protocol 052 study group. J Clin Oncol.

[3] Majumdar AK, Howard L, Goldberg MR, Hickey L, Constanzer M, Rothenberg PL (2006). Pharmacokinetics of aprepitant after single and multiple oral doses in healthy volunteers. J Clin Pharmacol.

[4] Bergstrom M, Hargreaves RJ, Burns HD, Goldberg MR, Sciberras D, Reines SA (2004). Human positron emission tomography studies of brain neurokinin 1 receptor occupancy by aprepitant. Biol Psychiatry.

[5] Muller RH, Peters K (1998). Nanosuspensions for the formulation of poorly soluble drugs: I. Preparation by a size reduction technique. Int J Pharm.

[6] Keck C, Kobierski S, Mauludin R, Muller RH (2008). Second generation of drug nanocrystals for delivery of poorly soluble drugs: Smartcrystal technology. Dosis.

[7] Attari Z, Kalvakuntla S, Reddy MS, Deshpande M, Rao CM, Koteshwara KB (2016). Formulation and characterisation of nanosuspensions of BCS class II and IV drugs by combinative method. J Exp Nano.

[8] Salazar J, Ghanem A, Muller RH, Moschwitzer JP (2012). Nanocrystals: Comparison of the size reduction effectiveness of a novel combinative method with conventional top-down approaches. Eur J Pharm Biopharm.

[9] Abdelwahed W, Degobert G, Stainmesse S, Fessi H (2006). Freeze-drying of nanoparticles: Formulation, process and storage considerations. Adv Drug Deliv Rev.

[10] Benjamin T, Rajyalakshmi Ch, Rambabu C (2013). Derivative spectrophotometric methods for determination of aprepitant in bulk and pharmaceutical formulation. Der Pharma Chem.

[11] Dissolution methods. U.S. Food and Drug Administration, U.S. Department of Health and Human Services. Available from: http://www.accessdata.fda.gov/scripts/cder/dissolution/dsp_SearchResults_Dissolutions.cfm.

[12] Katteboinaa S, Chandrasekhar VSRP, Balaji S (2009). Drug nanocrystals: A novel formulation approach for poorly soluble drugs. Int J Pharmtech Res.

[13] Shah VP, Lesko LJ, Fan J, Fleischer N, Handerson J, Malinowski H (1997). FDA Guidance for Industry: Dissolution Testing of Immediate Release Solid Oral Dosage forms. Dissolut Technol.

[14] Junyaprasert VB, Morakul B (2015). Nanocrystals for enhancement of oral bioavailability of poorly-water soluble drugs. Asian J Pharm Sci.

[15] Liu J, Zou M, Piao H, Liu Y, Tang B, Gao Y (2015). Characterization and pharmacokinetic study of aprepitant solid dispersions with soluplus(r). Molecules.

[16] Surolia R, Pachauri M, Ghosh PC (2012). Preparation and characterization of monensin loaded plga nanoparticles: In vitro anti-malarial activity against plasmodium falciparum. J Biomed Nanotechnol.

[17] Wu L, Zhang J, Watanabe W (2011). Physical and chemical stability of drug nanoparticles. Adv Drug Deliv Rev.

[18] Gao L, Zhang D, Chen M (2008). Drug nanocrystals for the formulation of poorly soluble drugs and its application as a potential drug delivery system. J Nanopart Res.

[19] Möschwitzer J, Lemke A. Method for carefully producing ultrafine particle suspensions and ultrafine particles and use thereof. WO/2006/108637. Unitied states patent US; 2006.

[20] Salazar J, Heinzerling O, Muller RH, Moschwitzer JP (2011). Process optimization of a novel production method for nanosuspensions using design of experiments (doe). Int J Pharm.

[21] Hu X, Chen X, Zhang L, Lin X, Zhang Y, Tang X (2014). A combined bottom-up/top-down approach to prepare a sterile injectable nanosuspension. Int J Pharm.

